# Nicotine Use Among Young Women Before Pregnancy: National Register‐Based Time Trend Analysis

**DOI:** 10.1111/1471-0528.70153

**Published:** 2026-01-07

**Authors:** Alma Larsdotter Zweygberg, Stamatia Tsampa, Rosaria Galanti, Cecilia Magnusson, Viktor H. Ahlqvist

**Affiliations:** ^1^ Department of Global Public Health Karolinska Institutet Solna Sweden; ^2^ Institute of Environmental Medicine Karolinska Institutet Solna Sweden

**Keywords:** epidemiology: perinatal, substance misuse in pregnancy, nicotine

The sharp decline in cigarette smoking is one of the major public health achievements of recent decades [1]. Tobacco companies have, however, responded to the increasingly effective tobacco control policies by marketing alternative nicotine delivery systems, including nicotine or tobacco pouches [1]. Following aggressive youth‐targeted and gender‐specific promotion, these products have gained popularity among adolescents and young adults, particularly girls and young women [1, 2]. Considering this new trend, any prenatal health risks associated with nicotine or tobacco pouches could be of considerable public health concern. Sweden occupies a unique position in this context with its longstanding tradition of tobacco pouch use (‘Swedish snus’), and a national exemption from the European Union–wide ban on its sale. While the Swedish snus is banned from sale, the oral nicotine pouches first introduced in 2016 are available in many European Union countries, the United States and elsewhere. Tobacco smoking entails clear and well‐established risks, whereas pouches, while lacking combustion by‐products, often deliver nicotine at doses exceeding those of cigarettes and contain other bioactive compounds [2]. The nicotine uptake is about 1 mg per cigarette [3], whereas the average nicotine uptake from a 6 mg strength nicotine pouch is 3.5 mg [4]. The nicotine uptake from nicotine pouches is larger than that from tobacco pouches [4]. The health impact of prenatal nicotine or tobacco pouches use, however, remains largely unknown [5]. In this brief research letter, we aim to describe the current time trends of nicotine or tobacco pouch use 3 months prior to pregnancy in the Swedish population.

Sweden has systematically tracked the use of ‘snus’ during preconception and pregnancy through national health registers. The term ‘snus’ is used for both the internationally marketed white nicotine pouches and the brown moist smokeless tobacco product used mainly in Scandinavia. Since the late 1990s, the Swedish Medical Birth Register (MBR) [6] has recorded self‐reported data on smoking and nicotine or tobacco pouches use in pregnancies carried beyond 22 (or 28 before 2008) gestational weeks. Becoming (near) nationwide in 2014, the Swedish Pregnancy Register [7] (SPR) has become a complementary source of pregnancy information, including all pregnancies for which there has been a first antenatal care visit (typically around 6–8 weeks of gestation), regardless of the pregnancy outcome and gestational week of delivery. These sources have been described in detail elsewhere [6, 7]. These sources thus offer a rare opportunity to monitor trends in prenatal nicotine or tobacco pouches. By combining the two registries, we analysed a near‐complete national cohort of pregnancies during 1999–2024. At the first antenatal visit (typically at 6–8 weeks of gestation), individuals retrospectively reported their use of cigarettes and nicotine or tobacco pouches 3 months before pregnancy. We included all pregnancies identifiable through the MBR or SPR, excluding overlapping pregnancies identified in both sources (pregnancy within the same woman ±8 months), and excluded those with missing tobacco or nicotine pouch use 3 months prior to pregnancy. We estimated the prevalence (100 × probability), and 95% confidence interval, using logistic regression and subsequent standardisation/G‐formula. The denominator for the prevalence is the number of pregnancies captured by MBR or SPR in a given year.

Based on registry data on 3 075 235 pregnancies, we here report a sharp rise in the use of nicotine or tobacco pouches during the preconception period (Figure 1). Between 1999 and September 2024, we observed a relative increase of 538% in the use of nicotine or tobacco pouches 3 months before pregnancy (from 1.6% [95% CI, 1.5, 1.8] to 10.2% [95% CI, 10.0, 10.4]). To our knowledge, these trends represent the first documented instance globally in which the use of nicotine or tobacco pouches has surpassed cigarette smoking during the preconception period.

**FIGURE 1:**
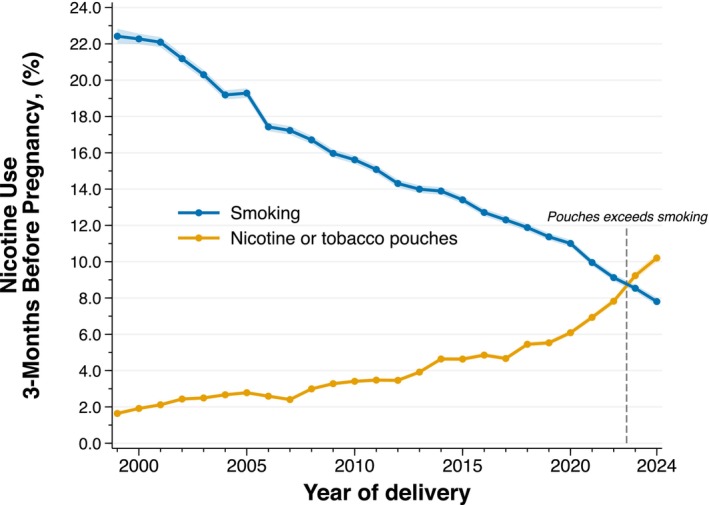
Annual prevalence, and 95% confidence intervals, of self‐reported nicotine or tobacco pouch use and cigarette smoking 3 months before pregnancy, Sweden, 1999–September 2024.

These findings signal a fundamental shift in preconception nicotine use that is likely to extend into the prenatal period. Although we could not distinguish between tobacco pouches and nicotine pouches in our data, the trend clearly shifted following the introduction of nicotine pouches marketing in 2016. Although existing evidence links prenatal nicotine exposure to adverse fetal development [5], the long‐term effects of high‐dose, sustained nicotine delivery from pouches remain unknown. Further research is needed to determine how often individuals discontinue use upon pregnancy recognition and whether cessation mitigates potential harm. Clinicians should recognise that pouches now account for a growing share of prenatal nicotine exposure and ensure that screening and counselling explicitly address their use. Targeted surveillance, expanded research and responsive policy measures are urgently required to guide clinical practice and protect maternal and child health.

## Author Contributions

V.H.A. led the conceptualisation of the study, supervised the project, and contributed to statistical analysis, figure creation and writing. S.T. contributed to conceptualisation, performed statistical analyses and created the figure. A.L.Z. drafted the manuscript and contributed to conceptualisation. R.G. contributed to interpretation of findings and critically revised the manuscript. C.M. contributed to interpretation, critical revision and writing. All authors approved the final version and agree to be accountable for all aspects of the work.

## Funding

This study was funded by the Swedish Research Council (Vetenskapsrådet), grant number (PI Magnusson, 2023‐02167).

## Ethics Statement

Ethical approval permitted by Swedish Ethical Review Authority on 2021‐01‐25, reference number 2020‐05516.

## Conflicts of Interest

The authors declare no conflicts of interest.

## Data Availability

The data used in this study are derived from Swedish national health registries. Due to ethical and legal restrictions under our approved ethical permit (Swedish Ethical Review Authority, reference number 2020‐05516), individual‐level data cannot be shared publicly. Data access is limited to the context of approved research publications or other outputs where no individual can be identified.
